# Large dipole moment induced efficient bismuth chromate photocatalysts for wide-spectrum driven water oxidation and complete mineralization of pollutants

**DOI:** 10.1093/nsr/nwz198

**Published:** 2019-12-02

**Authors:** Xianjie Chen, Yuan Xu, Xinguo Ma, Yongfa Zhu

**Affiliations:** 1 Department of Chemistry, Tsinghua University, Beijing 100084, China; 2 School of Science, Hubei University of Technology, Wuhan 430068, China

**Keywords:** bismuth chromate, dipole moment, internal electric field, water oxidation, complete mineralization

## Abstract

Herein, a wide-spectrum (∼678 nm) responsive Bi_8_(CrO_4_)O_11_ photocatalyst with a theoretical solar spectrum efficiency of 42.0% was successfully constructed. Bi_8_(CrO_4_)O_11_ showed highly efficient and stable photocatalytic water oxidation activity with a notable apparent quantum efficiency of 2.87% (420 nm), superior to many reported wide-spectrum driven photocatalysts. Most remarkably, its strong oxidation ability also enables the simultaneous degradation and complete mineralization for phenol, and its excellent performance is about 23.0 and 2.9 times higher than CdS and P25-TiO_2_, respectively. Its high activity is ascribed to the giant internal electric field induced by its large crystal dipole, which accelerates the rapid separation of photogenerated electron–hole pairs. Briefly, the discovery of wide-spectrum bismuth chromate and the mechanism of exponentially enhanced photocatalytic performance by increasing the crystal dipole throw light on improving solar energy conversion.

## INTRODUCTION

The conversion and utilization of solar energy for chemical fuel production and environmental remediation through artificial photocatalysis have been recognized to be an ideal route to address critical energy and environmental concerns [1–3]. The full utilization of solar light is a great challenge for achieving sufficient efficiency in practical applications. In the early stages, UV light-activated materials, such as TiO_2_, SrTiO_3_, NaTaO_3_, etc., dominated photocatalysis study, due to the wide bandgap of conventional semiconductors and their strong redox capability of charge carriers for igniting chemical reactions [4–7]. Nonetheless, the extremely low ratio of UV photons in solar energy greatly hinders the ability to maximize the solar-to-chemical energy conversion efficiency. In recent years, a number of mixed-anion and non-oxide materials such as (oxy)nitrides and (oxy)sulfides have been developed as attractive broadband light-responsive photocatalysts [8–11]. The valence band maximums (VB) of the mixed-anion materials can be substantially regulated by hybridization of O 2p or other introduced anion orbitals, enabling both broadband light absorption and suitable band potentials for both reduction and oxidation of water [12,13]. For example, BaNbO_2_N, reported by Hisatomi *et al*., could broaden the light absorption up to 740 nm and simultaneously shows efficient water oxidation [14]. However, narrowing the bandgap of a photocatalyst weakens the driving force for redox reactions, especially water oxidation and pollutant degradation, because these reactions involve a complicated multi-electron process [15]. Therefore, the development of wide-spectrum responsive and highly efficient photocatalysts for water oxidation and pollutant degradation is a critical issue to be addressed at present.

Bi-based oxometallate materials, such as BiVO_4_, Bi_2_WO_6_, Bi_2_MoO_6_, etc_._, have been widely studied as visible-light active photocatalysts, due to their high stability, abundant resources and low toxicity [16–19]. Moreover, they also exhibit excellent photocatalytic performance in water oxidation, which is mainly benefiting from their sufficiently deep VB position as compared to the potential for water oxidation and pollutant degradation [20,21]. In particular, the BiVO_4_ photocatalysts present highly efficient and stable water oxidation performance, and its highest solar-to-hydrogen energy conversion efficiency of 1.2% for Z-scheme pure-water splitting by coupling with SrTiO_3_: La, Rh has been reported [22]. Nevertheless, their relatively wide bandgaps (about 2.5 eV) greatly limit their further application. Recently, research into Cr-based layered double hydroxide photocatalysts revealed that the hybridization of Cr 3d orbitals with O 2p orbitals in [CrO_6_] clusters shifts the conduction band minimum (CB) down and results in wide visible-light absorption [23,24]. Inspired by the above, the construction of bismuth chromate photocatalyst may be a desired route to achieve wide-spectrum driven, efficient, and stable photocatalytic performance.

In this work, a wide-spectrum responsive Bi_8_(CrO_4_)O_11_ photocatalyst was successfully constructed. Owing to the hybridization of Cr 3d with O 2p orbitals shifting the conduction band minimum down, Bi_8_(CrO_4_)O_11_ allows its absorption up to the entire visible region (∼678 nm) with a theoretical solar spectrum efficiency of 42.0%. Moreover, attributed to the giant internal electric field (IEF) induced by its large dipole moment, Bi_8_(CrO_4_)O_11_ realized evidently rapid separation of photogenerated electron–hole pairs, thus showed highly efficient photocatalytic water oxidation activity with a notable apparent quantum yield of 2.87% (420 nm), superior to many reported wide-spectrum driven photocatalysts. Most remarkably, its strong oxidation ability also enables simultaneous degradation and complete mineralization for phenol, and its excellent performance is about 23.0 and 2.9 times higher than CdS and P25–TiO_2_, respectively.

## RESULTS AND DISCUSSION

**Figure 1.**
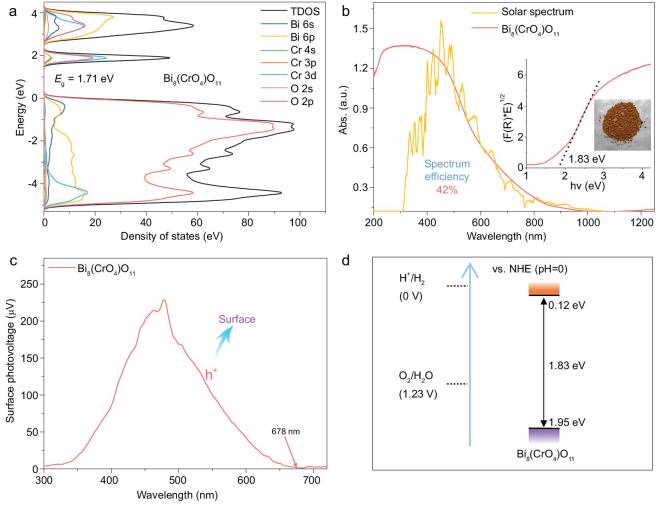
(a) The calculated density of state, (b) UV–vis–NIR DRS (the corresponding Tauc plots and a sample photograph appear in the inset), (c) the surface photovoltage spectrum, and (d) a schematic drawing of the redox potentials of Bi_8_(CrO_4_)O_11_.

Herein, monoclinic Bi_8_(CrO_4_)O_11_ nanorods (Figs S1–S3), a novel bismuth chromate photocatalyst, were successfully synthesized via a facile hydrothermal reaction. Then, density functional theory (DFT) was applied to calculate the electronic structure of this bismuth chromate. As shown in Fig. 1a, Bi_8_(CrO_4_)O_11_ possesses a relatively small bandgap of 1.71 eV. Moreover, the density of state of Bi_8_(CrO_4_)O_11_ reveals that its VB is mainly composed of O 2p and Bi 6s orbitals, in agreement with other Bi-based oxometallate photocatalysts, which could effectively avoid the self-oxidative deactivation by photogenerated holes. Also, its CB is mainly provided by the hybridization of Cr 3d orbitals with O 2p orbitals, demonstrating that the introduction of the [CrO_4_] cluster plays a crucial role in extending absorption into the entire visible region. Moreover, the indirect band structure of Bi_8_(CrO_4_)O_11_ is also confirmed by its electronic band diagram (Fig. S4a), which is in favor of confining the recombination of photogenerated electron–hole pairs. In the diffuse reflectance spectrum (DRS) (Fig. 1b), Bi_8_(CrO_4_)O_11_ nanorod photocatalyst displays a quite broad absorption band, practically allowing light absorption up to the entire visible region, and its highest theoretical solar utilization could reach 42.0%. Almost consistent with the above DFT result, the bandgap of Bi_8_(CrO_4_)O_11_ was calculated as 1.83 eV by the Kubelka–Munk function, which absolutely satisfies the thermodynamic energy criterion of water splitting [25,26]. As shown in Fig. 1c, Bi_8_(CrO_4_)O_11_ presents an evidently high surface photovoltage (SPV), and the response range could be extended to about 678 nm, demonstrating its wide-spectrum driven photocatalytic activity. Besides, it exhibits a positive surface photovoltage signal, meaning that the photogenerated holes are the main carriers and transfer to the surface to oxidize reactants. Therefore, the above results indicate that the Bi_8_(CrO_4_)O_11_ nanorod is a very promising wide-spectrum driven and stable photocatalyst.

Considering that the photocatalytic redox ability mainly depends on the energy band potential, the redox potentials of the Bi_8_(CrO_4_)O_11_ nanorod photocatalyst were calculated according to the DRS and Mott–Schottky plots (Fig. S5a) [27,28]. As shown in Fig. 1d, the CB of Bi_8_(CrO_4_)O_11_ is located at 0.12 eV vs. NHE (pH = 0), a little deeper than the reduction potential of H^+^/H_2_. Also, its VB of 1.95 eV is more positive than the oxidation potential of OH^−^/O_2_, which indicates that the photogenerated holes of Bi_8_(CrO_4_)O_11_ nanorod photocatalyst possess extremely strong oxidation capability, and can split water to release O_2_, and even completely mineralize organic pollutants under visible light.

**Figure 2.**
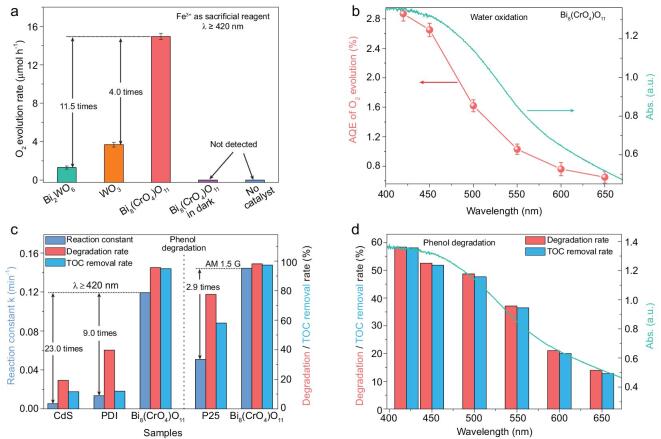
(a) A comparison of photocatalytic water oxidation activity over different photocatalysts. (b) The wavelength-dependent AQE of water oxidation over Bi_8_(CrO_4_)O_11_. (c) A comparison of degradation rate constants, degradation rates and TOC removal rates of phenol over different photocatalysts. (d) The wavelength-dependent degradation rate and TOC removal rate of phenol over Bi_8_(CrO_4_)O_11_.

We first evaluate the photocatalytic water oxidation performance over Bi_8_(CrO_4_)O_11_ nanorods. Figure 2a shows a comparison of the photocatalytic O_2_ evolution rate over different samples. It can be seen that Bi_8_(CrO_4_)O_11_ exhibited far superior photocatalytic water oxidation performance, and its average O_2_ evolution rate reached 14.94 μmol h^−1^, about 11.5 and 4.0 times higher than that of Bi_2_WO_6_ nanosheets [29] and commercial WO_3_ nanoparticles. Besides, Bi_8_(CrO_4_)O_11_ consequently achieved a considerable apparent quantum efficiency (AQE) 2.87% at 420 nm, even 0.65% at 650 nm (Fig. 2b), higher than many reported wide-spectrum driven photocatalysts (Table S2). In addition, the trend of AQE values for water oxidation over Bi_8_(CrO_4_)O_11_ is also consistent with its UV–vis DRS, further confirming that the photocatalytic water oxidation reaction is driven by its absorbed incident photons. Furthermore, after loading Co(OH)_2_ as co-catalyst, its photocatalytic water oxidation performance was improved by 2.1 times (Fig. S6). Just as importantly, no notable deactivation emerged over Bi_8_(CrO_4_)O_11_ during a continuous photocatalytic water oxidation reaction for 72 h (Fig. S7a**)**. By comparing its XRD patterns and XPS results before and after reaction (Figs S7b and S8), it could be found that the crystal structure and composition of Bi_8_(CrO_4_)O_11_ after reaction show no marked change, further indicating its robust resistance to water and light corrosion.

Most noticeably, the excellent activity of Bi_8_(CrO_4_)O_11_ is also manifested in photocatalytic degradation of phenol. As shown in Fig. 2c, Bi_8_(CrO_4_)O_11_ showed a superior photocatalytic degradation performance for phenol under visible light, and its degradation reaction constant could reach 0.119 min^−1^, about 22.5 and 8.8 times higher than CdS nanowires [30] and N,N′-di(propanoic acid)-perylene-3,4,9,10-tetracarboxylic diimide (PDI) supramolecular [31,32] photocatalysts, respectively. Even its degradation activity is not inferior to P25 TiO_2_ under simulated sunlight, being about 2.9 times higher than the latter. Remarkably, Bi_8_(CrO_4_)O_11_ also presented extremely strong mineralization ability, which almost enables simultaneous degradation and complete mineralization for phenol. The total organic carbon (TOC) removal rates of phenol over Bi_8_(CrO_4_)O_11_ under visible light and simulated sunlight are 94.8% (degradation rate: 95.5%) and 97.3% (degradation rate: 98.1%) in 0.5 h, respectively, while that of CdS, PDI and P25 are significantly lower than their corresponding degradation rates. In particular, even under 650 nm red light irradiation, Bi_8_(CrO_4_)O_11_ is still able to simultaneously degrade and completely mineralize phenol (Fig. 2d), and few wide-spectrum driven photocatalysts can achieve that [33]. Besides, Bi_8_(CrO_4_)O_11_ also exhibited highly efficient photocatalytic formaldehyde degradation activity under visible light in a continuous-flow system, and the removal rate could be maintained at about 95% (Fig. S10c). No notable deactivation emerges during continuous measurement for 76 h.

**Figure 3.**
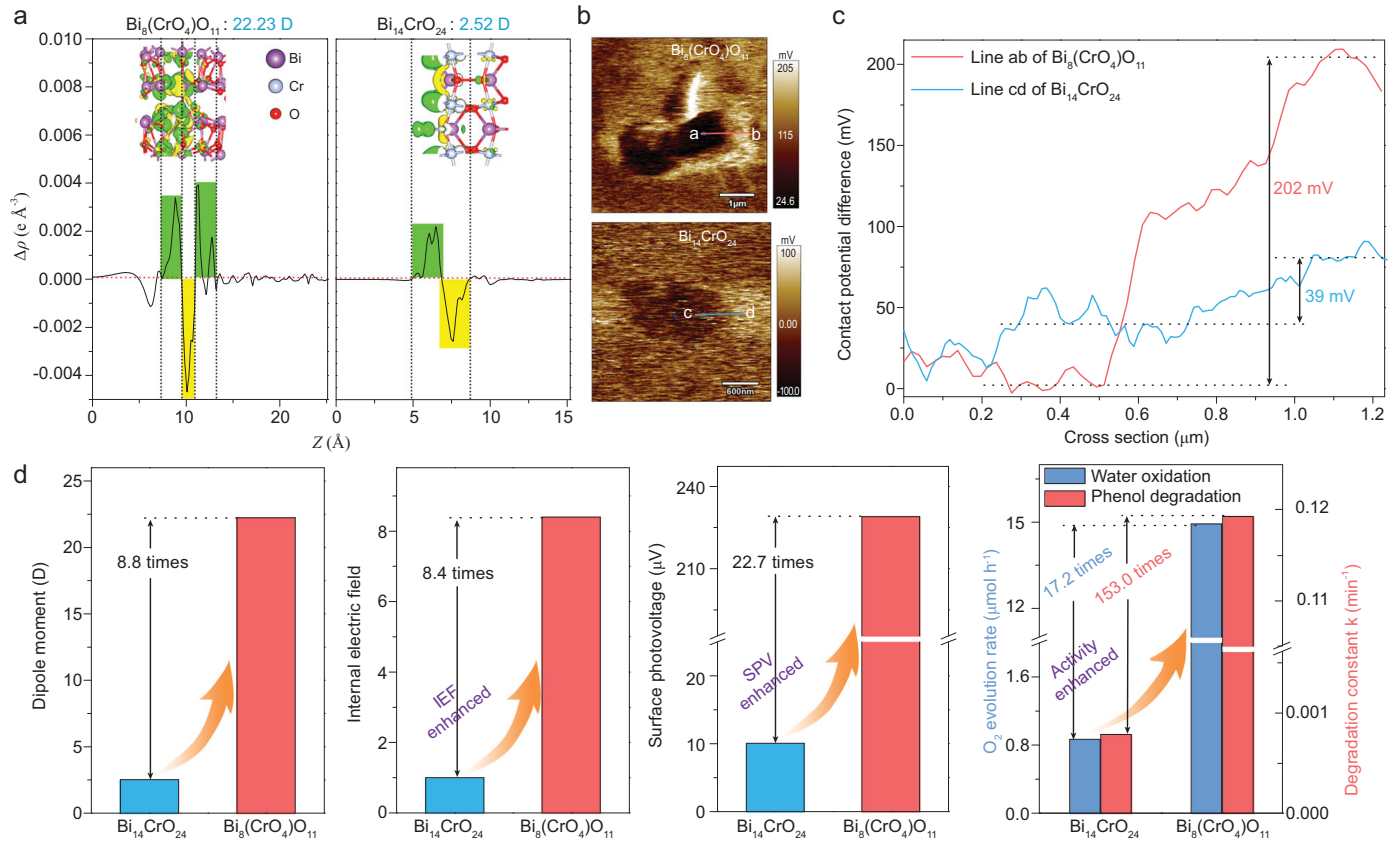
(a) A side view of the charge density difference and planar-averaged electron density difference Δρ(*z*) (the yellow and green areas indicate electron depletion and accumulation, respectively), (b) the surface potential image in the dark state, (c) the cross section of the surface potential distribution along the blue line ab and cd in (b) and (d) the correlation between dipole moments, internal electric field intensity, surface photovoltage and photocatalytic activity of Bi_8_(CrO_4_)O_11_ and Bi_14_CrO_24_.

It is well known that photocatalytic activity is closely related to the separation efficiency of photogenerated electron–hole pairs [34–36]. Previous studies have demonstrated that the IEF induced by the crystal dipole is considered to effectively boost the separation of photogenerated electron–hole pairs and enhance the photocatalytic performance exponentially, such as in Bi_2_MoO_6_, BiPO_4_, and BiOCl [37–39]. Therefore, to reveal the high activity mechanism of Bi_8_(CrO_4_)O_11_, the crystal dipoles of Bi_8_(CrO_4_)O_11_ and tetragonal Bi_14_CrO_24_ nanosheets (Figs S1 and S2) and their influence on the charge carrier separation and photocatalytic activity were studied. Through the Debye equation, the dipole moments of Bi_8_(CrO_4_)O_11_ and Bi_14_CrO_24_ were calculated to be 22.32 and 2.52 Debye (D), respectively. As shown in Fig. 3a, due to the existence of the great dipole of Bi_8_(CrO_4_)O_11_, the distortion of [BiO*_x_*] and [CrO*_y_*] polyhedrons induced an apparently uneven distribution of the electronic cloud between Bi–O and Cr–O, thus resulting in a giant IEF. Then, Kelvin probe force microscopy techniques were employed to reveal the IEF distribution in Bi_8_(CrO_4_)O_11_ and Bi_14_CrO_24_. As shown in Fig. 3b, Bi_8_(CrO_4_)O_11_ shows an obvious difference in the contact potential difference (CPD) between the edge and the bulk, about 202 mV (Fig. 3c), but the CPD difference over Bi_14_CrO_24_ is virtually invisible, only about 39 mV. According to the literature, the relatively large CPD difference between the two regions reflects that a relatively strong IEF is formed in the crystal [40–42], consequently demonstrating the existence of a greater IEF in Bi_8_(CrO_4_)O_11_.

**Scheme 1.**
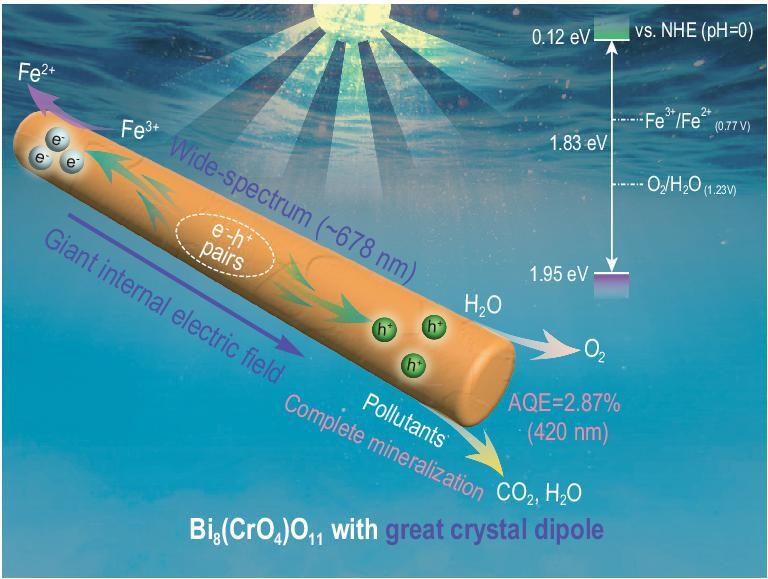
Schematic mechanism of the photocatalytic reaction over Bi_8_(CrO_4_)O_11_.

Furthermore, the intensity of their IEF was measured via the model developed by Kanata-Kito *et al*. (details are given in the supplementary data online) [43,44]. It can be found that the IEF of Bi_8_(CrO_4_)O_11_ is 8.4 times as high as that of Bi_14_CrO_24_ (Fig. S13), well consistent with the above results. Benefiting from its greater IEF, Bi_8_(CrO_4_)O_11_ presented an evidently stronger surface photovoltage response and photocurrent density (Fig. S16), about 22.7 and 4.0 times higher than Bi_14_CrO_24_, respectively, revealing that a faster charge carrier transfer kinetics emerges in Bi_8_(CrO_4_)O_11_. As expected, Bi_8_(CrO_4_)O_11_ exhibited 17.2 and 153.0 times higher photocatalytic water oxidation and degradation performance than Bi_14_CrO_24_, respectively. Then, after summarizing the above results into Fig. 3d, it can be found that the IEF, charge separation efficiency and photocatalytic activity of bismuth chromate are positively correlated with their dipole moments; thus Bi_8_(CrO_4_)O_11_ with a greater dipole showed a significantly higher IEF, charge separation efficiency and photocatalytic performance. Therefore, as illustrated in Scheme 1, the large crystal dipole of Bi_8_(CrO_4_)O_11_ induces a giant IEF, which accelerates the rapid separation of photogenerated electron–hole pairs and exponentially enhances its photocatalytic performance. Most importantly, based on the above mechanism, many more efficient photocatalysts can be designed successfully by regulating the crystal dipole.

## CONCLUSION

In conclusion, a wide-spectrum (∼678 nm) responsive Bi_8_(CrO_4_)O_11_ nanorod photocatalyst was constructed via the hybridization of Cr 3d with O 2p orbitals. Attributed to the giant IEF induced by its large dipole moment, Bi_8_(CrO_4_)O_11_ realizes evidently rapid separation of photogenerated electron–hole pairs, thus showing highly efficient photocatalytic water oxidation performance with a notable apparent quantum yield of 2.87% (420 nm), superior to many reported wide-spectrum driven photocatalysts. Most remarkably, its strong oxidation ability also enables simultaneous degradation and complete mineralization for phenol, and its excellent performance is about 23.0 and 2.9 times higher than CdS and P25-TiO_2_, respectively. Briefly, the discovery of wide-spectrum bismuth chromate and the mechanism of exponentially enhanced photocatalytic performance by increasing the crystal dipole throw light on designing efficient wide-spectrum photocatalysts.

## METHODS

### Synthesis of samples

NaBiO_3_ and other chemicals were purchased from Aladdin and Sigma-Aldrich, respectively, and used without further purification. For Bi_8_(CrO_4_)O_11_ nanorods, 0.56 g NaBiO_3_ was ultrasonically dispersed in 80 mL deionized water, followed by addition of 7.35 mL 25 mmol L^−1^ Cr(NO)_3_ aqueous solution under vigorous stirring. Then the resulting solution was transferred into a 100 mL Teflon-lined stainless autoclave and maintained at 180°C for 6 h. The brown-red Bi_8_(CrO_4_)O_11_ was collected by centrifuge separation, rinsed thoroughly with ethanol and deionized water several times, and dried at 70°C overnight. For Bi_14_CrO_24_ nanosheets, 1 mmol Bi(NO_3_)_3_ and 0.084 mmol Cr(NO_3_)_3_ were ultrasonically dissolved in a certain concentration of mannitol aqueous solution (25 mL), followed by addition of 5 ml saturated Na_2_CO_3_ solution under vigorous stirring. Then the resulting solution was transferred into a 50 mL Teflon-lined stainless autoclave and maintained at 150°C for 12 h. The precursor was collected by centrifuge separation, rinsed thoroughly with ethanol and deionized water several times, and dried at 70°C overnight. The precursor was then calcined in a crucible at a certain temperature for 10 min under air atmosphere to yield orange-red Bi_14_CrO_24_ nanosheets.

For comparison, BiWO_6_ nanoplates were synthesized as in [29], and WO_3_ nanoparticles were purchased from Aladdin.

### Characterization

XRD patterns of the samples were obtained on a Rigaku D/max-2400 X-ray diffractometer using Cu Kα1 (λ = 0.154 18 nm) at 40 kV and 200 mA, with a scan step of 0.02°. The morphologies of the samples were measured by transmission electron microscopy (TEM) on a Hitachi HT 7700 at an accelerating voltage of 100 kV and high-resolution transmission electron microscopy (HRTEM) on a JEOL JEM-2100F operated at an accelerating voltage of 200 kV. Field emission scanning electron microscopy (FESEM) on a Hitachi SU-8010 was used to further investigate the morphology. XPS measurements were performed using an ESCALAB 250Xi instrument (Thermo Scientific) with Al Kα radiation. DRS were obtained on a Cary 5000 (Varian) with BaSO_4_ as a reference. The surface potential images of the samples were measured by Kelvin probe force microscopy (KPFM) in ambient atmosphere on a Cypher VRS (Oxford Instruments) and a Pt/Ir-coated Si tip was used as a Kelvin tip. The surface photovoltage measurements were conducted with a home-built instrument as previously reported [45]. Photoelectrochemical measurements were performed on a CHI660E electrochemical workstation, using a standard three-electrode cell with a working electrode, a Pt-wire counter electrode and a saturated calomel reference electrode. Na_2_SO_4_ (0.1 mol L^−1^) was used the electrolyte solution. The working electrode was prepared by dip-coating photocatalyst slurry on ITO glass electrode (2 × 4 cm^2^).

### Photocatalytic performance evaluation

The photocatalytic water oxidation reaction under visible-light irradiation was performed in a Pyrex top-irradiation reaction vessel with a stationary temperature at 5°C, which was connected to a glass closed gas system (Labsolar-6A, PerfectLight). 100 mg photocatalyst was suspended individually in 100 mL aqueous solution (pH = 2.5) containing 10 mmol L^−1^ Fe(NO_3_)_3_ as a sacrificial reagent. The suspension was then thoroughly degassed and irradiated using a 300 W Xe lamp with a cut-off filter (λ ≥ 420 nm, light intensity 250–260 mW cm^−2^). The evolved gases were analyzed at given time intervals by an online gas chromatograph (GC-2002 N/TFF, TCD detector, Ar carrier, 5 Å molecular sieve column).

The AQE for water oxidation was measured using a 300 W Xe lamp (FX300, PerfectLight) with different band-pass filters of 420, 450, 500, 550, 600, and 650 nm (FWHM = 15 nm). The irradiation area was controlled as 1.2 × 1.2 cm^2^. The average intensity was determined by an optical power meter (S310C connected to a PM100D console, Thorlabs). The AQE was calculated as follows:

$$\begin{eqnarray*}
{{\rm{AQE\ }}}\nonumber\\
&=&\frac{{4{\rm{\ }} \times {\rm{\ the\ number\ of\ evolved\ }}{{\rm{O}}_2}{\rm{\ molecules}}}}{{{\rm{the\ number\ of\ incident\ photons}}}}{\rm{\ }}\nonumber\\
&& \times {\rm{\ }}100{\rm{\% }}.
\end{eqnarray*}$$

The photodegradation reactions were carried in quartz tube reactors with a 50 mL 10 ppm phenol pollutant solution and 25 mg photocatalyst powders. The reaction solution was kept at 35°C by a recirculating cooling water system. The visible-light source was obtained from a 300 W Xe lamp with a cut-off filter (λ ≥ 420 nm). Before light irradiation, the suspension solutions were first ultrasonically dispersed for 5 min and then magnetically stirred for 1 h in the dark to reach adsorption–desorption equilibrium. At certain time intervals, a suspension (2 mL) was extracted and centrifuged to remove the photocatalysts. The concentration of phenol pollutants was determined by a high-performance liquid chromatography (HPLC) system (Shimadzu LC-20AT) with a C18 reversed-phase column, and the total organic carbon (TOC) in the aqueous solution was analyzed using a TOC analyzer (Multi N/C 2100, Analytik Jena AG).

## Supplementary Material

nwz198_Supplemental_File
