# Supplementary material for: Large dipole moment induced efficient bismuth chromate photocatalysts for wide-spectrum driven water oxidation and complete mineralization of pollutants
Source: Natl Sci Rev. 2019 Dec 2;7(3):652–9. doi: 10.1093/nsr/nwz198 (PMC8289005; doi:10.1093/nsr/nwz198)
Supplement: nwz198_Supplemental_File [file nwz198_supplemental_file.pdf]

Supplementary data

Large dipole moment induced efficient bismuth chromate photocatalysts for wide-spectrum driven water oxidation and complete mineralization of pollutants

Xianjie Chen,[1] Yuan Xu,[2] Xinguo Ma,[2] Yongfa Zhu[1,]*

[1]Department of Chemistry, Tsinghua University, Beijing, 100084, P. R. China

[2]School of Science, Hubei University of Technology, Wuhan 430068, P. R. China

*Corresponding author. E-mail: zhuyf@tsinghua.edu.cn

Table of Contents

1. Experimental section

1.1 Photocatalytic performance evaluation

1.2 Calculation methods

2. Results and discussion

2.1 Electronic band diagram

2.2 XRD, DRS and XPS measurement

2.3 Morphology measurement

2.4 Mott-Schottky plots

2.5 Photocatalytic water oxidation activity

2.6 Stability of photocatalyst

2.7 Photocatalytic Z-scheme water splitting

2.8 photocatalytic degradation performance

2.9 Dipole moment and internal electric field

2.10 Photoelectrochemical characterizations

3. Reference

1. Experimental section

1.1 Photocatalytic performance evaluation

The photocatalytic Z-scheme water splitting reaction with Fe^{3+}/Fe^{2+} redox mediator under visible-light irradiation was performed in a Pyrex top-irradiation reaction vessel with a stationary temperature at 5 °C, which was connected to a glass closed gas system (Labsolar-6A, PerfectLight). The Ru loaded $SrTiO_3$:Rh (0.10 g) synthesized as in reference [1] and $Bi_8(CrO_4)O_{11}$ (0.05 g) as H_2-evolving and O_2-evolving photocatalysts, respectively, were suspended in 100 mL 2 mmol L^{-1} $Fe(NO_3)_3$ aqueous solution. The pH value of the solution was adjusted to be 2.5 by adding a small amount of aqueous HNO_3. The suspension was then thoroughly degassed and irradiated using a 300 W Xe lamp with a cutoff filter (λ≥420 nm, light intensity 250-260 mW cm^{-2}). The evolved gases were analyzed at given times intervals by an online gas chromatograph (GC-2002 N/TFF, TCD detector, Ar carrier, 5Å molecular sieve column).

The solar-to-hydrogen (STH) conversion efficiency is given by:

$$STH = \frac{R(H_2) \times \Delta G_r}{P \times S} \times 100\%$$

Where $R(H_2)$, ΔG_r, P, and S represent the rate of hydrogen evolution, the Gibbs energy for the reaction (H_2O (l) → H_2 (g) + 1/2 O_2 (g)), the energy intensity of the AM 1.5G solar irradiation (100 mW/cm^2), and the irradiated sample area, respectively.

The gas-phase photocatalytic degradation of formaldehyde was conducted with a self-made flat-plate quartz reactor operated in a continuous-flow mode. A 300 W Xe lamp with a cutoff filter (λ≥420 nm) was employed as the light source. A 10 mg of as-prepared photocatalyst was spread evenly on a glass plate (area, 40 cm^{-2}). A bubbler that contained formaldehyde was immersed in an ice water bath and formaldehyde (about 25 ppm) bubbled with oxygen from the gas cylinder was fed to the catalyst at a total flow rate of 140 mL min^{-1}. The concentrations of formaldehyde was conducted by a photoacoustic spectrometer (GASER ONE).

1.2 Calculation methods

All theoretical calculations were performed by the generalized gradient approximation (GGA) within the Perdew-Burke-Emzerhof (PBE) [2] exchange-correlation functional implemented in the CASTEP code [3]. The ultrasoft pseudopential in the Vanderbilt form was used for description of ion-electron interaction [4]. The valence atomic configurations are $6s^2 6p^3$ for Bi, $3s^2 3p^6 3d^5 4s^1$ for Cr, $2s^2 2p^4$ for O, respectively. The plane-wave function was set with the cut-off energy of 500 eV. Here, a Monkhorst–Pack grid [5] of $8 \times 6 \times 8$ was used. Geometry optimizations were done before single point energy calculation with the self-consistent convergence accuracy of 1×10^{-6} eV atom^{-1}. The convergence criterion for the maximal force between atoms is 0.01 eV. The maximum displacement is 5×10^{-4} Å, and the stress is less than 0.02 GPa.

2. Results and discussion

2.1 XRD, DRS and XPS measurement

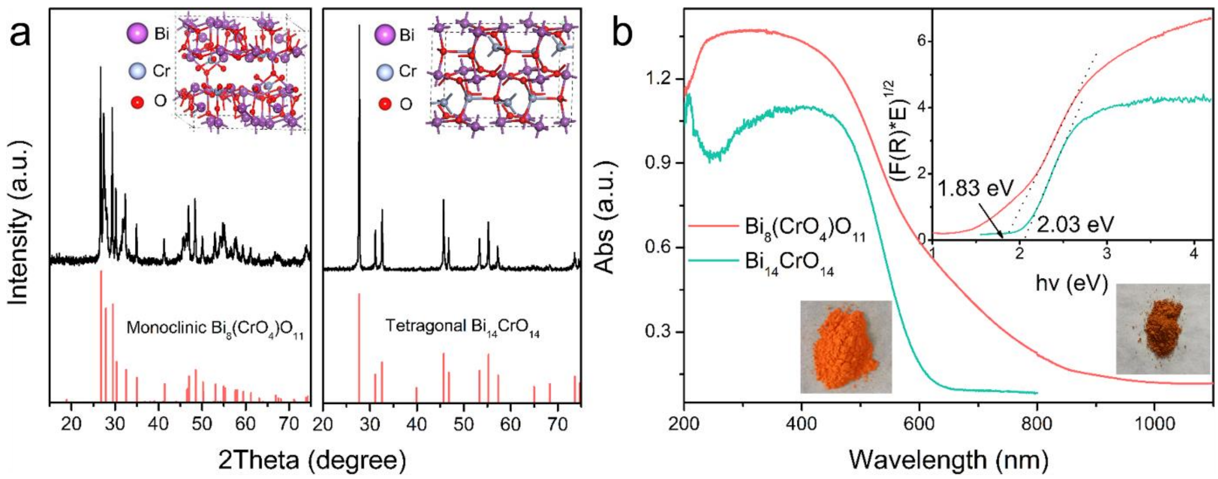

Figure S1. (a) XRD pattern (Schematic drawing of the crystal structure inset), and (b) UV-Vis DRS (The corresponding Tauc plots and sample photograph inset) of $Bi_8(CrO_4)O_{11}$ and $Bi_{14}CrO_{14}$.

As shown in Figure S1a, the XRD pattern of the as-prepared $Bi_8(CrO_4)O_{11}$ and $Bi_{14}CrO_{14}$ exhibit typical monoclinic and tetragonal phase, and the sharp diffraction peaks demonstrate their high crystallinity. Besides, in the UV-Vis-NIR DRS (Figure S1b), $Bi_8(CrO_4)O_{11}$ and $Bi_{14}CrO_{14}$ both display a broad absorption band, allowing its light absorption up to the entire visible region, and their band gap could be calculated to 1.83 and 2.03 eV by the Kubelka-Munk function, respectively, which absolutely satisfies the thermodynamic energy criterion of water splitting [6, 7].

The theoretical spectrum efficiency equals to the wavelength range of the material absorption spectrum divided by the measured solar spectrum. The solar spectrum was collected by Optical Fiber Spectrometer (AULTPP-P4000, CEAULIGHT). It should be pointed out that the absorption range of the material needs to be counted from the intrinsic absorption of the electronic transition and ended in the smaller wavelength in solar spectrum or absorption spectrum. So, for $Bi_8(CrO_4)O_{11}$, it should be 678-300 nm. For the measured solar spectrum, it is 1200-300 nm. Thus, the theoretical spectrum efficiency can be calculated as following equation [8].

$$\text{Theoretical spectrum efficiency} = \frac{Absorption\ range}{Collcted\ solar\ spectrum\ range} = \frac{678 - 300\ nm}{1200 - 300\ nm} = 42.0\%$$

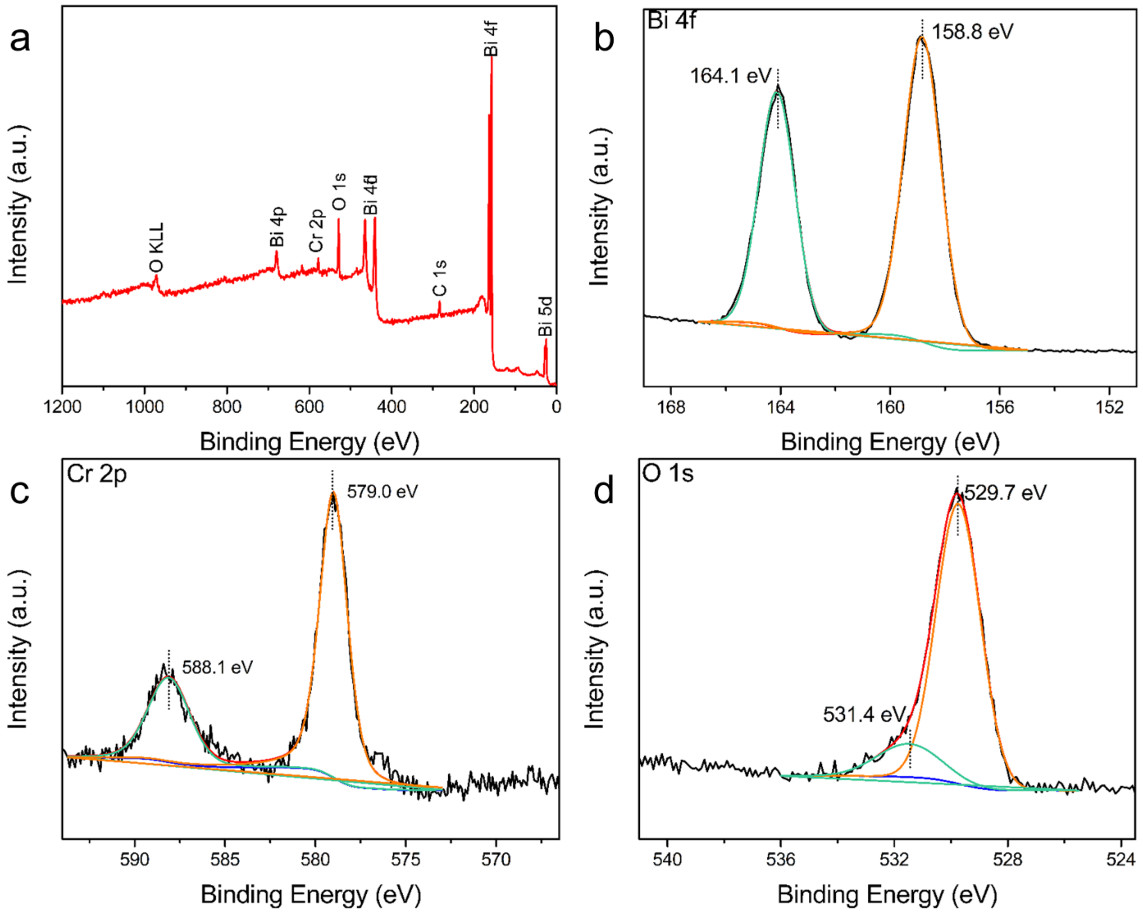

Figure S2. XPS spectra of $Bi_8(CrO_4)O_{11}$: (a) survey spectrum, (b) high resolution spectrum of Bi 4f, (c) Cr 2p, and (d) O 1s.

In the XPS spectra, the prepared $Bi_8(CrO_4)O_{11}$ sample is composed of Bi, Cr, and O. In Figure S2b, two peaks at 157.8 eV and 163.1 eV are attributed to Bi $4f_{7/2}$ and Bi $4f_{5/2}$, respectively. In the high resolution spectra of Cr 2p, two peaks at 577.9 eV and 587.0 eV are assigned to the oxidation state of +6 for Cr in the $Bi_8(CrO_4)O_{11}$.

2.2 Morphology measurement

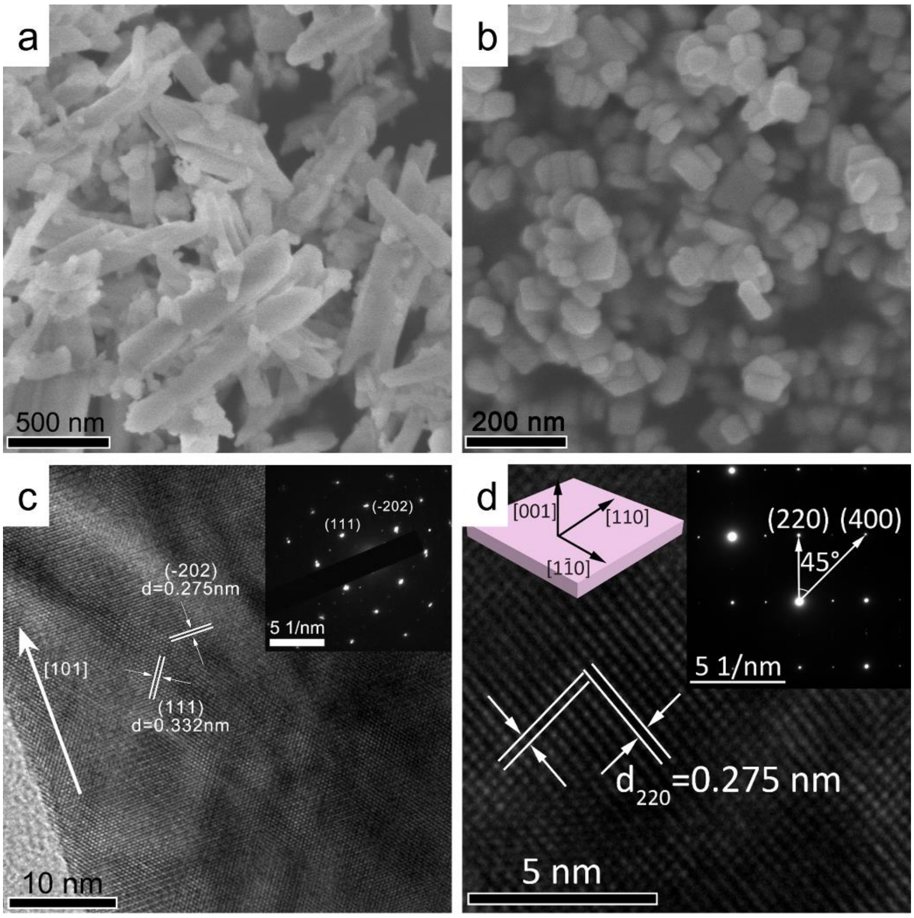

Figure S3. SEM image and HRTEM image (SAED pattern inset) of (a, c) $Bi_8(CrO_4)O_{11}$ and (b, d) $Bi_{14}CrO_{14}$.

As shown in the SEM image (Figure S3a), $Bi_8(CrO_4)O_{11}$ presents single-crystal nanorod structure with about 100 nm in diameter. The characteristic lattice fringe space of monoclinic phase $Bi_8(CrO_4)O_{11}$ (111) and (-202) is 0.332 and 0.275 nm, respectively, observed from its HRTEM image (Figure S3c). Based on the selected area electron diffraction (SAED) pattern, it could be inferred that $Bi_8(CrO_4)O_{11}$ nanorods grow along [101] axis. Moreover, the SEM images (Figure S3b) of $Bi_{14}CrO_{14}$ present its single crystal plate-shaped structure with a size of about 50 nm. Besides, it can be seen from the EDS mapping that the prepared $Bi_{14}CrO_{14}$ sample is composed of Bi, Cr, and O. And the clear lattice fringes in HRTEM image (Figure S3d) demonstrate the highly crystalline nature of $Bi_{14}CrO_{14}$ nanoplate, with an interplanar lattice spacing of 0.275 nm indexed to the (220) atomic planes.

Additionally, the angle labeled in the SAED pattern is 45°, which is consistent with the theoretical value of the angle between the (220) and (400) planes, as well as the set of diffraction spots could be indexed as the [001] zone axis of $Bi_{14}CrO_{14}$ nanoplate. In view of the above results and the symmetries of tetragonal $Bi_{14}CrO_{14}$, the bottom and top surface of nanoplate could be identified as {001} facets, while the four lateral surfaces are {110} facets.

2.3 Electronic band diagram

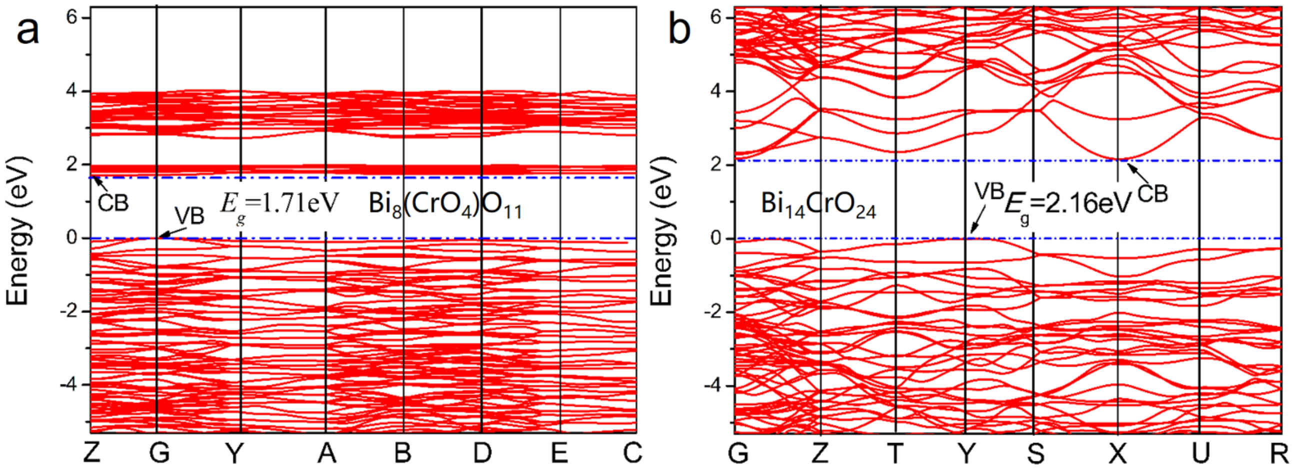

Figure S4. The calculated electronic band diagram of (a) $Bi_8(CrO_4)O_{11}$ and (b) $Bi_{14}CrO_{14}$.

As shown in Figure S4a, the indirect band structure of $Bi_8(CrO_4)O_{11}$ with the CB at Z point and VB at G point is also revealed, which is in favor of confining the recombination of photogenerated electron-hole pairs. Besides, it can be seen that the conduction band is more dispersion than valence band, indicating an easier of electron transfer from valence band [9]. And $Bi_{14}CrO_{24}$ also has the same electronic structure (Figure S4b).

2.4 Mott-Schottky plots

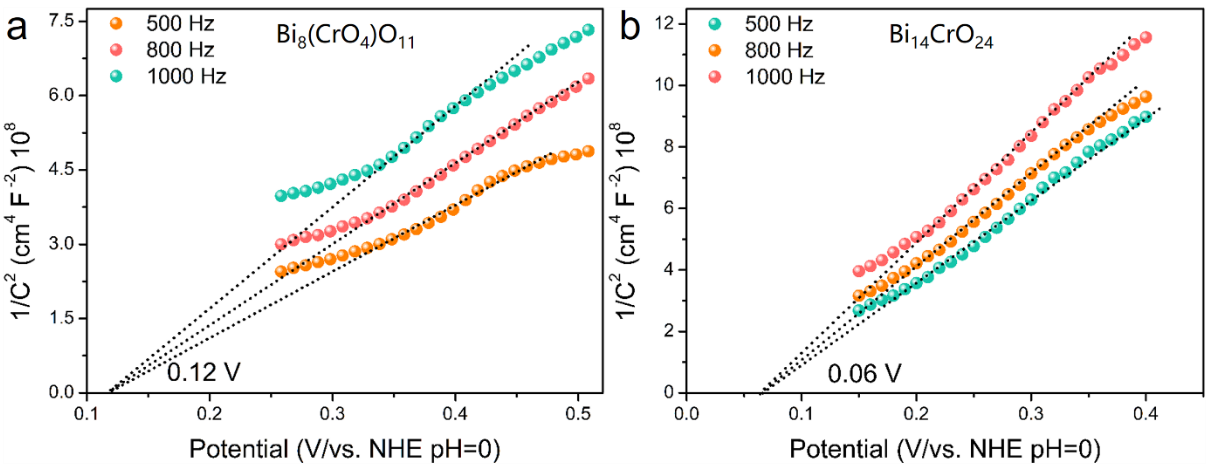

Figure S5. Mott-Schottky plots of (a) $Bi_8(CrO_4)O_{11}$ and (b) $Bi_{14}CrO_{14}$.

In the Mott-Schottky plots, the positive slopes demonstrate the n-type semiconductor characteristics of $Bi_8(CrO_4)O_{11}$ and $Bi_{14}CrO_{14}$. In general, for an n-type semiconductor, the flat-band potential is approximately at the conduction band potential [10]. Thus, we could conclude that the conduction band potentials of $Bi_8(CrO_4)O_{11}$ and $Bi_{14}CrO_{14}$ are 0.12 and 0.06 eV vs. NHE (pH=0), respectively.

2.5 Photocatalytic water oxidation activity

Table S1. Wavelength dependent AQE of photocatalytic water oxidation over $Bi_8(CrO_4)O_{11}$.[a]

Wavelength (nm)	420	450	500	550	600	650
O_2 evolution (μmol)	28.97	19.71	18.48	14.70	10.87	9.01
Light density (mW cm^{-2})	22.15	15.28	21.11	24.03	22.01	19.79
Irradiation area (cm^2)	1.44	1.44	1.44	1.44	1.44	1.44
Irradiation time (h)	10	10	10	10	10	10
AQE (%)	2.87	2.65	1.62	1.03	0.76	0.65

[a] Reaction condition: 100 mg $Bi_8(CrO_4)O_{11}$, 100 mL distilled water containing 10 mmol L^{-1} $Fe(NO_3)_3$ as sacrificial regent. All reaction were carried out at 278 K and 3 kPa.

λ=420 nm：

The number of incident photons:

$$\mathrm{N} = \frac{E\lambda}{hc} = \frac{22.15 \times 1.44 \times 10^{-3} \times 10 \times 3600 \times 650 \times 10^{-9}}{6.626 \times 10^{-34} \times 3 \times 10^{8}} = 2.43 \times 10^{21}$$

AQE:

$$\mathrm{AQE} = \frac{4 \times the\ number\ of\ evolved\ O_2\ molecules}{N} \times 100\%$$

$$= \frac{4 \times 6.02 \times 10^{23} \times 28.97 \times 10^{-6}}{2.43 \times 10^{21}} \times 100\% = 2.87\%$$

λ=650 nm：

The number of incident photons:

$$\mathrm{N} = \frac{E\lambda}{hc} = \frac{19.79 \times 1.44 \times 10^{-3} \times 10 \times 3600 \times 650 \times 10^{-9}}{6.626 \times 10^{-34} \times 3 \times 10^{8}} = 3.35 \times 10^{21}$$

AQE:

$$\mathrm{AQE} = \frac{4 \times the\ number\ of\ evolved\ O_2\ molecules}{N} \times 100\%$$

$$= \frac{4 \times 6.02 \times 10^{23} \times 9.01 \times 10^{-6}}{3.35 \times 10^{21}} \times 100\% = 0.65\%$$

Table S2. Summary of wide-spectrum driven photocatalysts for water oxidation activity

Photocatalysts	Band gap (eV)	Co-catalyst	Sacrificial agents	Light	O_2 evolution rate (umol g^{-1} h^{-1})	AQE	Ref.
$Bi_8(CrO_4)O_{11}$	1.83	None	10 mM $Fe(NO_3)_3$	λ≥420 nm	149.4	2.87% (420nm), 0.65% (650nm)	This work
$BaNbO_2N$	1.68	CoO_x	50 mM $AgNO_3$	λ≥410 nm	60.9	0.04% (640nm)	[11]
$BaZrO_3$-$BaTaO_2N$	1.75	IrO_2	10 mM $AgNO_3$	λ≥420 nm	77.0	0.03% (420nm)	[12]
$BaNb_{1-x}Ta_xO_2N$	1.82	CoO_x	10 mM $AgNO_3$	λ≥420 nm		0.24% (420nm)	[13]
$BaWO_xN_y$-$BaTaO_2N$	1.87	IrO_2	10 mM $AgNO_3$	λ≥420 nm	105.0		[14]
$Ba_5Ta_4O_{15-x}N_x$	1.96	None	10 mM $AgNO_3$	λ≥420 nm	16.0		[15]
$Sm_2Ti_2S_2O_5$ nanoplates	2.06	IrO_2	10 mM $AgNO_3$	λ≥420 nm	83.0	1.3% (420nm)	[16]
Nano-Fe_2O_3	2.06	None	20 mM $AgNO_3$	λ≥420 nm		0.61% (375nm)	[17]
$MgTa_2O_{6-x}N_x$	2.17	CoO_x	10 mM $AgNO_3$	λ≥420 nm	66.7		[18]
$SrTaO_2N$ polyhedra	2.18	CoO_x	50 mM $AgNO_3$	400-500 nm	15.0	1.6%(400-500nm)	[19]
$SrTaO_2N$ polycrystals	2.20	CoO_x	50 mM $AgNO_3$	400-500 nm	6.3	0.6%(400-500nm)	[19]
$Sr_5Ta_4O_{15-x}N_x$	2.20	None	10 mM $AgNO_3$	λ≥420 nm	16.0		[15]
$Ba(Mg_{1/3}Ta_{2/3})O_{3-x}N_y$	2.21	None	10 mM $AgNO_3$	λ≥420 nm	18.7		[20]
$Sr_2Ta_2O_{7-x}N_x$	2.21	None	10 mM $AgNO_3$	λ≥420 nm	12.0		[15]
Bi_4NbO_8Cl	2.39	None	5 mM $FeCl_3$	λ≥420 nm	42.0	0.4% (420nm)	[21]
$LiCuTa_3O_9$	2.48	CoPi	10 mM $AgNO_3$	λ≥420 nm		0.21% (420nm)	[22]

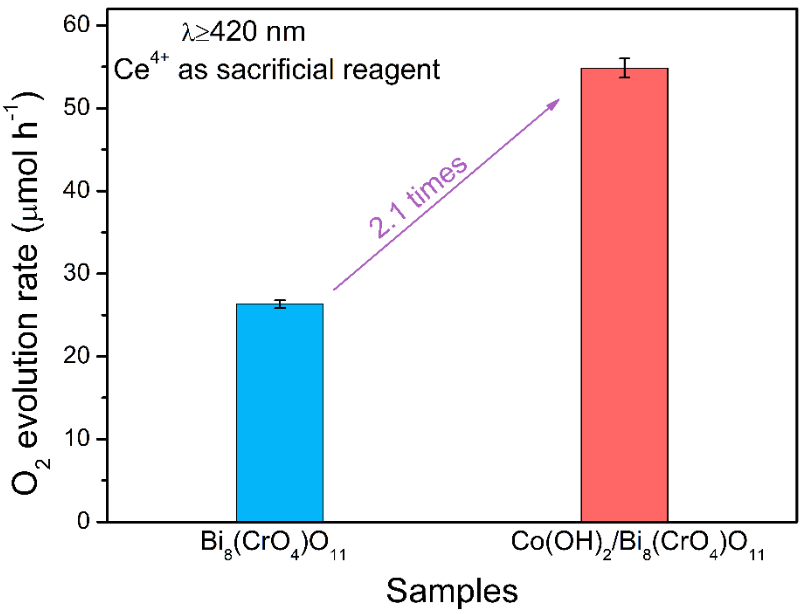

Figure S6. The comparison of photocatalytic water oxidation activity over $Bi_8(CrO_4)O_{11}$ and $Co(OH)_2/Bi_8(CrO_4)O_{11}$. Reaction condition: 100 mg samples; 100 mL 5 mmol L^{-1} $Ce(NH_4)_2(NO_3)_6$ aqueous solution; visible light (λ≥420 nm).

According to the previously reported literature[23], $Co(OH)_2/Bi_8(CrO_4)O_{11}$ with 3wt.% loading capacity was synthesized. Considering that $Co(OH)_2$ will dissolve in Fe^{3+} aqueous solution (pH=2.5), thus $Ce(NH_4)_2(NO_3)_6$ was used as the sacrificial agent to evaluate their photocatalytic water oxidation activity. As shown in Figure S6, the O_2 evolution rate of $Co(OH)_2/Bi_8(CrO_4)O_{11}$ reached 54.84 μmol h^{-1}, about 2.1 times higher than $Bi_8(CrO_4)O_{11}$.

2.6 Stability of photocatalyst

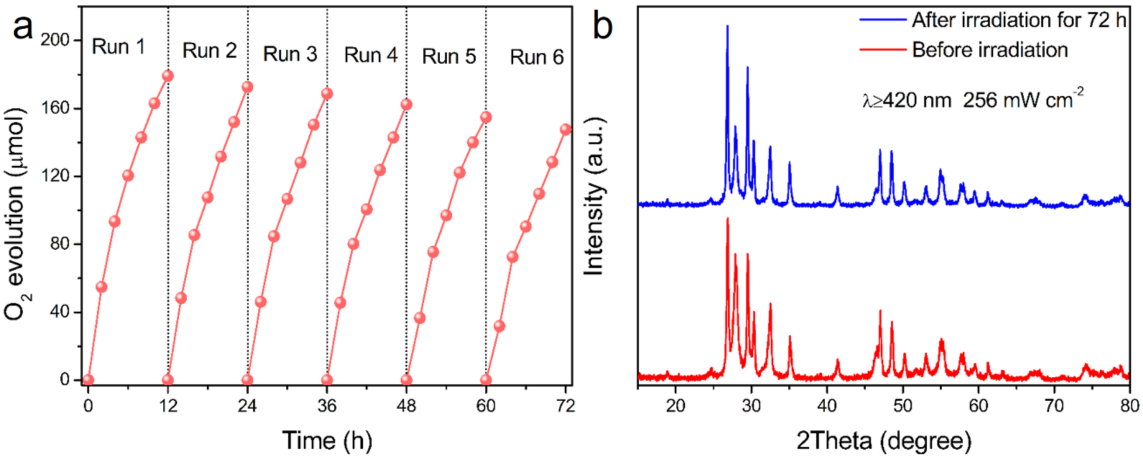

Figure S7. (a) The cycling stability test of photocatalytic water oxidation over $Bi_8(CrO_4)O_{11}$ and (b) XRD patterns of $Bi_8(CrO_4)O_{11}$ before and after the repeated cycles of water oxidation reaction.

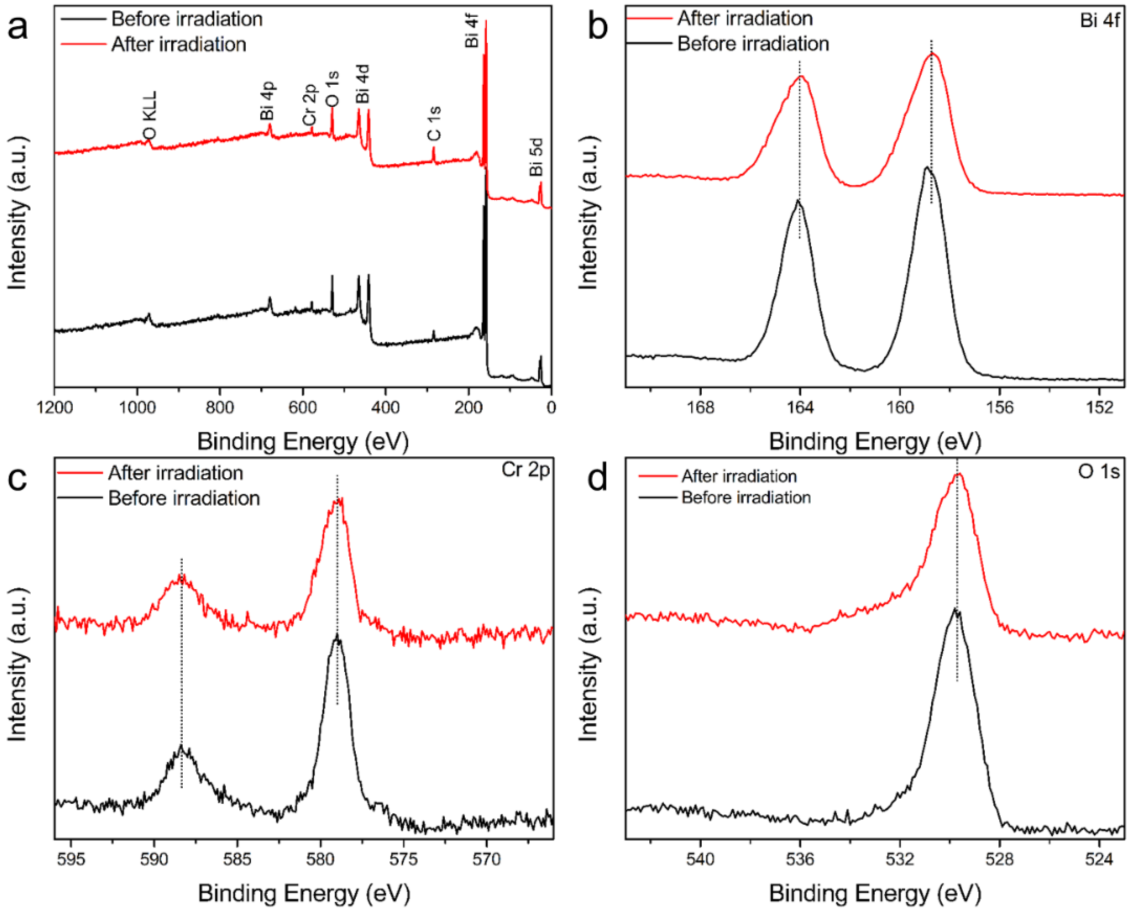

Figure S8. XPS spectra of $Bi_8(CrO_4)O_{11}$: (a) survey spectrum, (b) high resolution spectrum of Bi 4f, (c) Cr 2p, and (d) O 1s before and after the repeated cycles of water oxidation reaction.

According to the XRD pattern and XPS results (Figure S7b, S8), the crystal structure and composition of $Bi_8(CrO_4)O_{11}$ after water oxidation reaction show no remarked change, indicating its robust resistance to water and light corrosion.

2.7 Photocatalytic Z-scheme water splitting

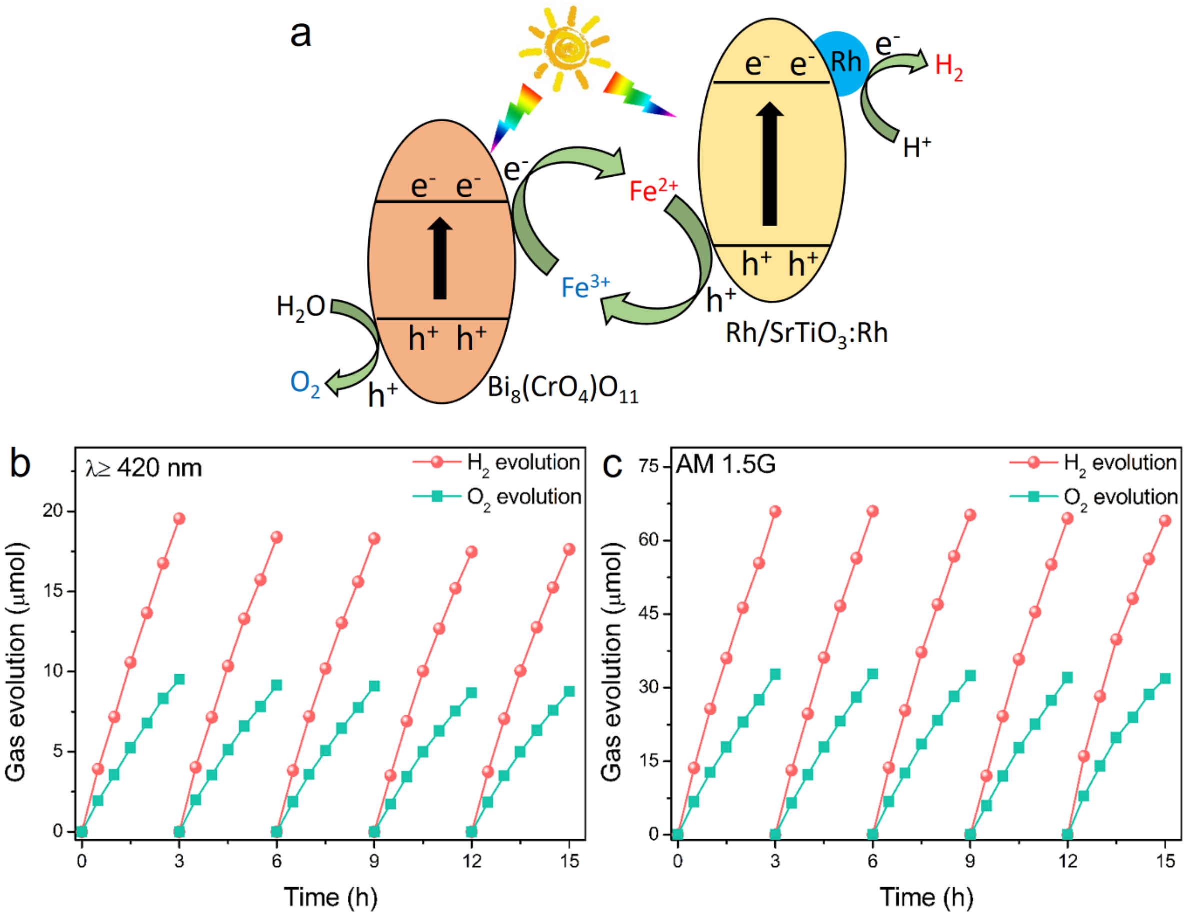

Figure S9. (a) The working diagram of Z-scheme water splitting over $Bi_8(CrO_4)O_{11}$ coupled with $Ru/SrTiO_3:Rh$ via Fe^{3+}/ Fe^{2+} redox mediator. The time course of Z-scheme water splitting under (b) visible light ($\lambda \geq 420$ nm) and (c) AM 1.5G simulated sunlight.

As shown in **Figure S9**a, a Z-scheme photocatalytic overall water splitting over $Bi_8(CrO_4)O_{11}$ coupled with $Ru/SrTiO_3:Rh$ as an H_2-evolving photocatalyst was successfully realized, involving a redox mediator of Fe^{3+}/Fe^{2+}. Under visible light irradiation, both H_2 and O_2 with an evolution rate of 4.32 and 2.12 μmol h^{-1} were generated simultaneously, respectively, quite close to the stoichiometric ratio of 2:1 (Figure S9b). Moreover, the gas evolution rate was increased to 14.37 and 7.15 μmol h^{-1}

for H_2 and O_2 under AM 1.5G simulated sunlight (Figure S9c), and the STH energy-conversion efficiency was further determined to be 3.7×10^{-3}% (Table S3). The excellent stability of this Z-scheme water splitting system was amply confirmed through a long-term reaction (for 30 h) with the periodical evacuation, and a total of 207.25 μmol O_2 were generated, absolutely exceeding the molar amount of $Bi_8(CrO_4)O_{11}$ (57.81 μmol) used for the reaction, which reveals that this reaction proceeded photocatalytically.

Table S3. STH of the Z-scheme water splitting over $Bi_8(CrO_4)O_{11}$ coupled with Ru/$SrTiO_3$:Rh via Fe^{3+}/ Fe^{2+} redox mediator under AM 1.5G simulated sunlight

H_2 evolution (μmol)	O_2 evolution (μmol)	Light intensity (mW/cm^2)	Irradiation area (cm^2)	Irradiation time (h)	STH (%)
0.2265	0.1105	100	1	4	0.0037

$$STH = \frac{R(H_2)\times \Delta G_r}{P\times S}\times 100\% = \frac{0.2265\times 10^{-6} \div (4\times 3600)\times 237130}{100\times 1\times 10^{-3}}\times 100\% = 0.0037\%$$

2.8 Photocatalytic degradation performance

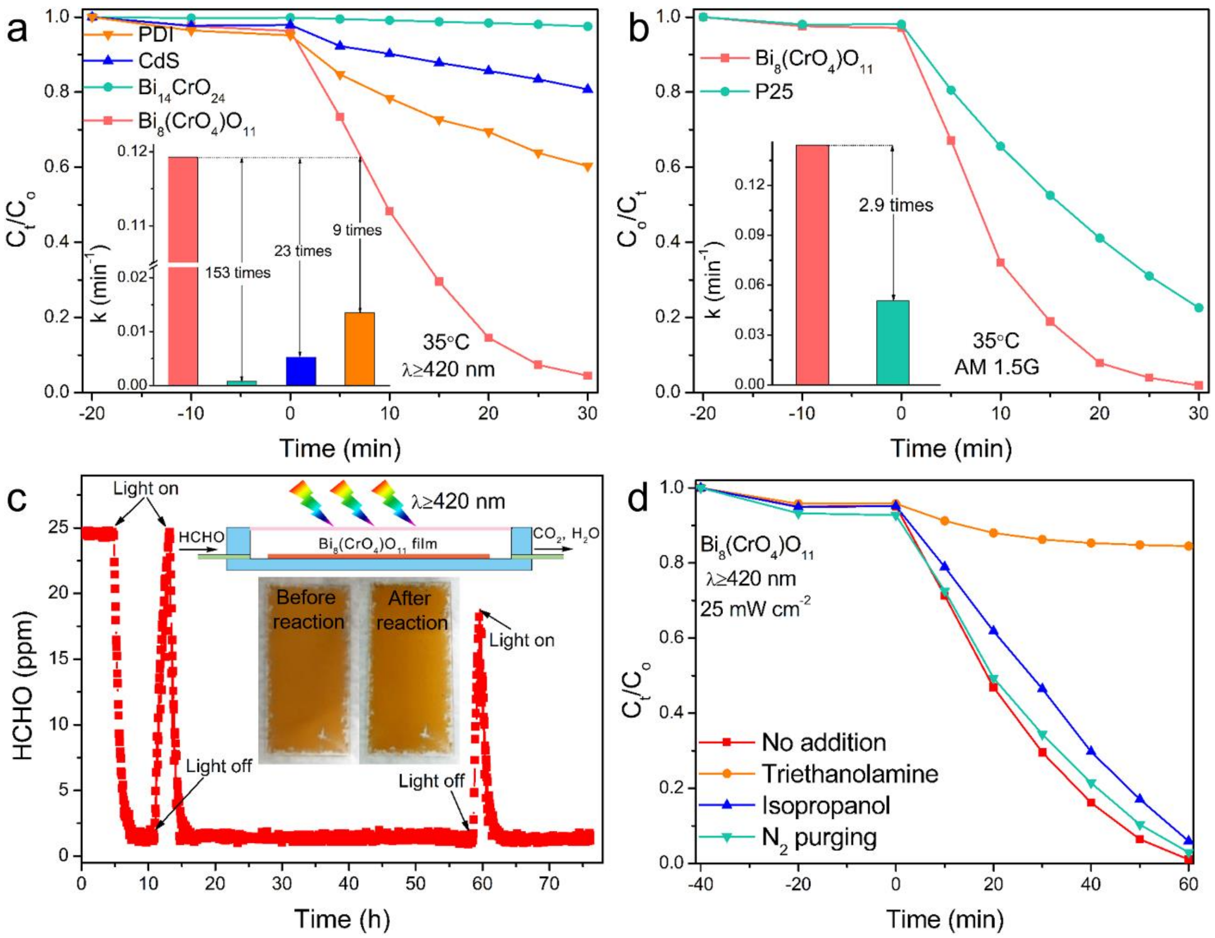

Figure S10. The photocatalytic degradation of phenol over different photocatalysts under (a) visible light (λ≥420 nm) and (b) AM 1.5G simulated sunlight. (c) The photocatalytic degradation HCHO over $Bi_8(CrO_4)O_{11}$ under visible light (λ≥420 nm). (d) The photocatalytic degradation of phenol over $Bi_8(CrO_4)O_{11}$ under visible light (λ≥420 nm) in the presence of different sacrificial agent.

Figure S10c shows the photocatalytic degradation HCHO over $Bi_8(CrO_4)O_{11}$ under visible light. It can be seen that $Bi_8(CrO_4)O_{11}$ exhibits highly efficient photocatalytic performance, and the removal rate of HCHO could reach 95%. Besides, no notable deactivation emerges during a continuous measurement for 76 h. The trapping experiment revealed that the active species of $Bi_8(CrO_4)O_{11}$ is mainly photogenerated holes (Figure S10d).

2.9 Dipole moment and internal electric field

Through the Debye equation of μ = neR, the net dipole of [BiO_x] and [CrO_y] units were calculated. Here, μ is the net dipole moment in Debye (10^{-18} esu cm), n is the total number of electrons, e is the charge of electron (-4.8×10^{-10} esu), and R is the difference (cm) between the "centroids" of the positive and negative charge, calculated by the following equation:

$$R=\sqrt{\left(\frac{\sum_{i=1}^{n} x_i}{n}-x\right)^2+\left(\frac{\sum_{i=1}^{n} y_i}{n}-y\right)^2+\left(\frac{\sum_{i=1}^{n} z_i}{n}-z\right)^2}$$

Thus, The geometric center coordinates of [BiO_5], [BiO_4], [BiO_3] and [CrO_4] polyhedrons of $Bi_8(CrO_4)O_{11}$ after optimized are (7.085, 6.706, 9.426), (2.864, 9.025, 1.953), (7.527, 4.744, 5.824) and (3.513, 3.009, 6.401), respectively. According to the Debye equation, the dipole moment of the [BiO_5], [BiO_4], [BiO_3] and [CrO_4] polyhedrons in a unit cell are calculated to be 6.01 D, 7.00 D, 9.15 D and 0.07 D (Figure S11a), respectively. Thus, the dipole moment in a unit cell of $Bi_8(CrO_4)O_{11}$ is 22.23 D.

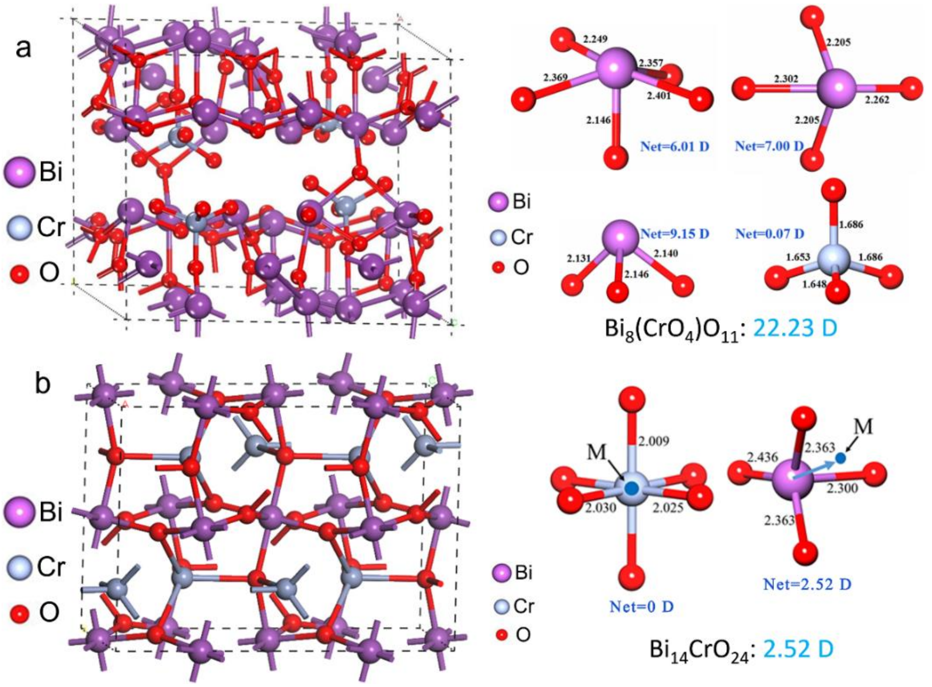

Figure S11. The crystal structure and dipole moment of [BiO_x] and [CrO_y] polyhedrons in (a) $Bi_8(CrO_4)O_{11}$ and (b) $Bi_{14}CrO_{24}$.

Then, as for $Bi_{14}CrO_{24}$, the geometric center coordinates of $[BiO_4]$ tetrahedron after optimized is (2.88681, 1.93697, 2.56248). The dipole moment of the $[BiO_4]$ tetrahedron in a unit cell is verified to be 2.52 D by calculation and is along the (-0.23293, 0, -0.20481) direction. The geometric centers of positive and negative charge coincide in $[CrO_6]$ octahedron, and the dipole moment of the $[CrO_6]$ octahedron in a unit cell is verified to be 0 D (Figure S11b). Thus, the dipole moment in a unit cell of $Bi_{14}CrO_{24}$ is 2.52 D.

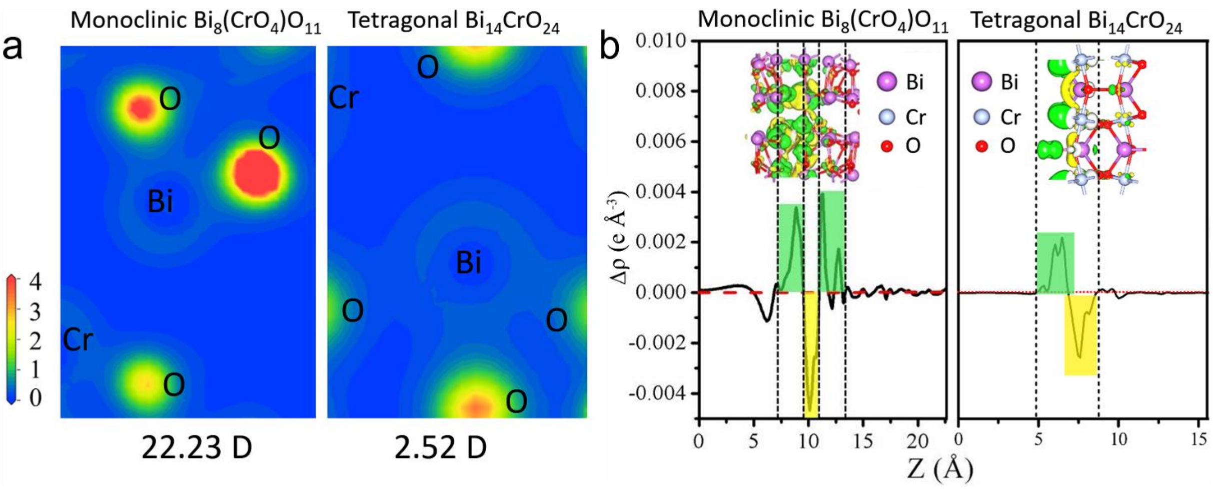

Figure S12. (a) The charge density contour plots viewed from (011) facet of $Bi_8(CrO_4)O_{11}$ and (001) facet of $Bi_{14}CrO_{24}$. (b) The side view of the charge density difference and planar-averaged electron density difference $\Delta\rho(z)$ of $Bi_8(CrO_4)O_{11}$ and $Bi_{14}CrO_{24}$, the yellow and green areas indicate electron depletion and accumulation, respectively.

Due to the existence of dipole moment in the $Bi_8(CrO_4)O_{11}$ crystal cell, the distortion of $[BiO_5]$, $[BiO_4]$, $[BiO_3]$ and $[CrO_4]$ polyhedrons would change the distribution of the electronic cloud between Bi-O and Cr-O. It can be seen from the charge density contour plots (Figure S12a) and the charge density difference (Figure S12b), the electron clouds mainly gather around the $[BiO_x]$ layer, while the density of the electron clouds around the $[CrO_4]$ layer is significantly lower. Therefore, a giant IEF in $Bi_8(CrO_4)O_{11}$ could be induced due to the uneven distribution of charge density between [BiOx] and $[CrO_4]$ layers [24]. However, the little difference of charge distribution around $[BiO_x]$ and $[CrO_4]$ layers is observed owing to the small dipole moment in $Bi_{14}CrO_{24}$, which leads to a smaller IEF.

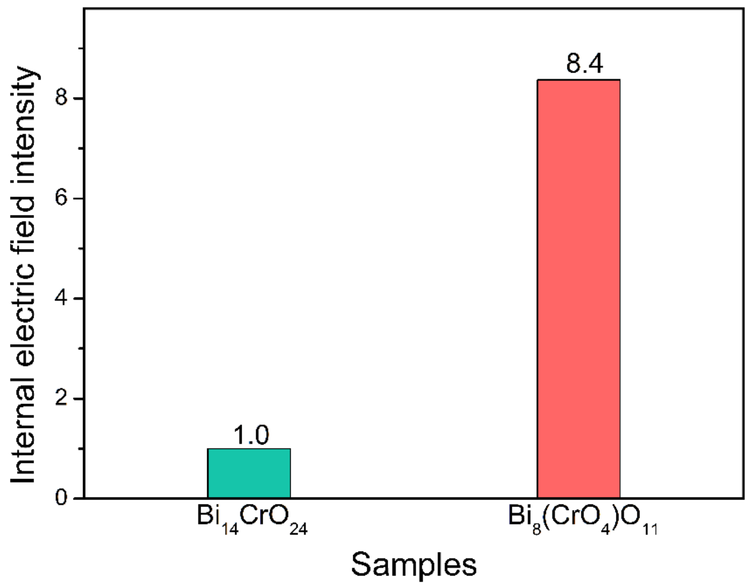

Figure S13. The internal electric field intensity of $Bi_8(CrO_4)O_{11}$ and $Bi_{14}CrO_{24}$ ((assuming the intensity of $Bi_{14}CrO_{24}$ to be “1”).

The IEF magnitude of the $Bi_8(CrO_4)O_{11}$ and $Bi_{14}CrO_{24}$ was calculated by using the following equation developed by Kanata et al.[25-28] It can be found that the IEF of $Bi_8(CrO_4)O_{11}$ is 8.4 times as high as that of $Bi_{14}CrO_{24}$ (Figure S13).

$$Fs=(-2V_s\rho/\varepsilon\varepsilon_0)^{1/2}$$

Where Fs is the internal electric field magnitude, Vs is the surface voltage, ρ is the surface charge density, Ɛ is the low-frequency dielectric constant, and $Ɛ_0$ is the permittivity of free space. The above equation reveals that the internal electric field magnitude is mainly determined by the surface voltage and the charge density because Ɛ and $Ɛ_0$ are two constants. In order to evaluate the internal electric field magnitude of $Bi_8(CrO_4)O_{11}$ and $Bi_{14}CrO_{24}$, we carefully figured out their charge densities by the transient photocurrent density measurements and surface voltages by open-circuit potentials measurements, which are shown in Figure S14 and Figure S15, respectively.

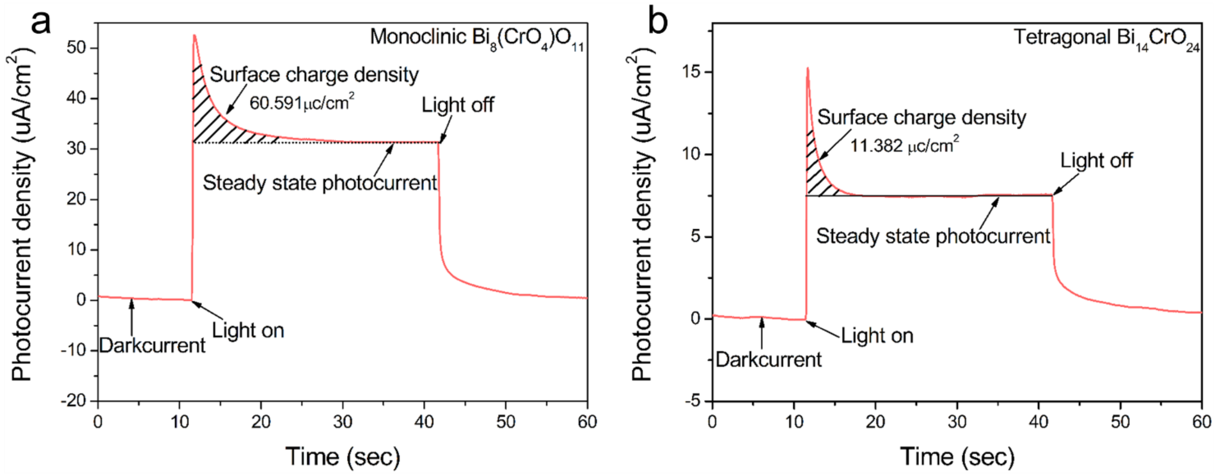

Figure S14. The transient photocurrent density of (a) $Bi_8(CrO_4)O_{11}$ and (b) $Bi_{14}CrO_{24}$.

Le Formal and Gratzel et al. have reported that, by integrating the measured transient photocurrent density minus the steady state values of photocurrent with respect to time, the value is proportional to the number of positive charges accumulated at the surface [29]. Here, we measure the transient photocurrent and integrate the transient anodic photocurrent peaks. The surface charge density of $Bi_8(CrO_4)O_{11}$ and $Bi_{14}CrO_{24}$ is shown in Figure S14.

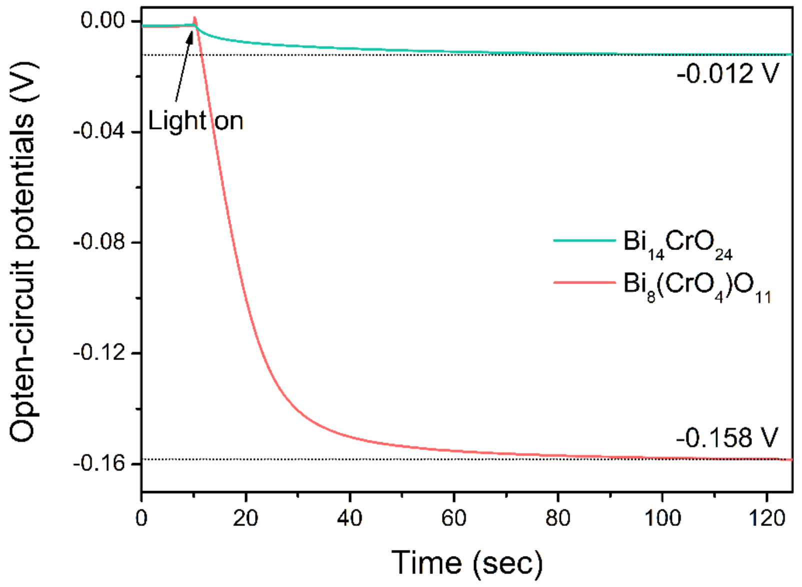

Figure S15. The open-circuit potentials of $Bi_8(CrO_4)O_{11}$ and $Bi_{14}CrO_{24}$.

2.10 Photoelectrochemical characterizations

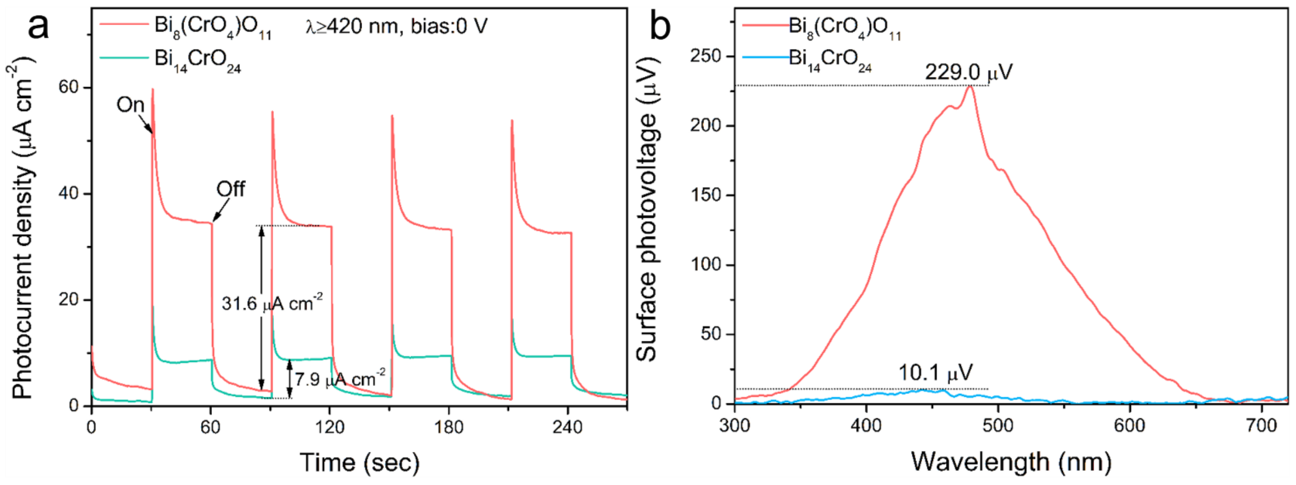

Figure S16. (a) The photocurrent density and (b) surface photovoltage spectrum of $Bi_8(CrO_4)O_{11}$ and $Bi_{14}CrO_{24}$.

As shown in Figure S16a, $Bi_8(CrO_4)O_{11}$ shows a significantly stronger photocurrent response of 31.6 μA/cm^2, about 4 times higher than $Bi_{14}CrO_{24}$, revealing that a faster charge carrier transfer kinetics emerges in $Bi_8(CrO_4)O_{11}$. As shown in Figure S16b, $Bi_8(CrO_4)O_{11}$ presents an evidently higher surface photovoltage (SPV), which indicates that a lower recombination rate of electron-hole pairs occurs over $Bi_8(CrO_4)O_{11}$. And the response range could be extended to about 678 nm, demonstrating its wide-spectrum driven photocatalytic activity. Besides, they both exhibit positive surface photovoltage signals, meaning that photogenerated holes are the main carriers and transfer to the surface to oxidize reactants.

Table S4. The bond lengths of [BiO_5], [BiO_4], [BiO_3] and [CrO_4] polyhedrons in monoclinic $Bi_8(CrO_4)O_{11}$ and its atom sites

	Atom	Atomic Population (Milliken)	x (Å)	y (Å)	z (Å)	Bond	Bond Length (Å)
[BiO_5]	Bi	1.56	6.409	6.861	9.020		
	O(1)	-0.93	5.856	6.968	11.20	Bi-O(1)	2.249
	O(2)	-0.62	6.960	9.026	9.810	Bi-O(2)	2.369
	O(3)	-0.91	8.550	6.965	9.136	Bi-O(3)	2.149
	O(4)	-0.94	7.148	5.801	6.996	Bi-O(4)	2.401
	O(5)	-0.91	6.910	4.771	9.990	Bi-O(5)	2.357
[BiO_4]	Bi	1.49	3.280	9.026	2.839		
	O(1)	-0.93	0.981	9.026	2.955	Bi-O(1)	2.302
	O(2)	-0.91	2.674	7.263	1.662	Bi-O(2)	2.205
	O(3)	-0.59	5.127	9.026	1.533	Bi-O(3)	2.262
	O(4)	-0.91	2.674	10.79	1.662	Bi-O(4)	2.205
[BiO_3]	Bi	1.44	6.382	4.945	5.190		
	O(1)	-0.92	8.409	5.423	4.671	Bi-O(1)	2.146
	O(2)	-0.93	7.024	3.009	5.804	Bi-O(2)	2.131
	O(3)	-0.94	7.148	5.801	6.996	Bi-O(3)	2.140
[CrO_4]	Cr	0.64	3.513	3.009	6.401		
	O(1)	-0.62	4.307	1.636	6.973	Cr-O(1)	1.686
	O(2)	-0.91	3.565	3.009	4.754	Cr-O(2)	1.648
	O(3)	-0.62	4.307	4.381	6.973	Cr-O(3)	1.686
	O(4)	-0.91	1.956	3.009	6.958	Cr-O(4)	1.653

Table S5. The bond lengths of [BiO_4] tetrahedron and [CrO_6] octahedron in tetragonal $Bi_{14}CrO_{24}$ and its atom sites

	Atom	Atomic Population (Milliken)	x (Å)	y (Å)	z (Å)	Bond	Bond Length (Å)
[BiO_4]	Bi	1.69	3.11974	1.93697	2.76729		
	O(1)	-0.75	5.48909	1.93697	2.20063	Bi-O(1)	2.436
	O(2)	-0.75	1.69321	3.53296	3.76897	Bi-O(2)	2.363
	O(3)	-0.75	2.67172	1.93697	0.511350	Bi-O(3)	2.300
	O(4)	-0.75	1.69321	0.340978	3.76897	Bi-O(4)	2.363
[CrO_6]	Cr	0.64	5.63475	3.87395	8.13593		
	O(1)	-0.62	5.48915	1.93698	7.62455	Cr-O(1)	2.030
	O(2)	-0.91	7.32799	3.53304	9.19294	Cr-O(2)	2.009
	O(3)	-0.62	6.75888	4.21486	6.48097	Cr-O(3)	2.025
	O(4)	-0.91	5.78035	5.81093	8.64730	Cr-O(4)	2.030
	O(5)	-0.62	3.94151	4.21486	7.07891	Cr-O(5)	2.009
	O(6)	-0.91	4.51062	3.53304	9.79088	Cr-O(6)	2.025

3. References

1. Tao X, Zhao Y, Mu L et al. Bismuth Tantalum Oxyhalogen: A Promising Candidate Photocatalyst for Solar Water Splitting. *Adv Energy Mater* 2018; **8**: 1701392.
2. Perdew JP, Burke K, Ernzerhof M. Generalized gradient approximation made simple. *Phys Rev Lett* 1996; **77**: 3865.
3. Segall M, Lindan PJ, Probert Ma et al. First-principles simulation: ideas, illustrations and the CASTEP code. *J Phys Condens Mat* 2002; **14**: 2717.
4. Vanderbilt D. Soft self-consistent pseudopotentials in a generalized eigenvalue formalism. *Phys Rev B* 1990; **41**: 7892.
5. Monkhorst HJ, Pack JD. Special points for Brillouin-zone integrations. *Phys Rev B* 1976; **13**: 5188.
6. Chen S, Takata T, Domen K. Particulate photocatalysts for overall water splitting. *Nat Rev Mater* 2017; **2**: 17050.
7. Kong D, Zheng Y, Kobielusz M et al. Recent advances in visible light-driven water oxidation and reduction in suspension systems. *Mater Today* 2018; **21**: 897-924.
8. Zhang Z, Zhu Y, Chen X et al. A Full-Spectrum Metal-Free Porphyrin Supramolecular Photocatalyst for Dual Functions of Highly Efficient Hydrogen and Oxygen Evolution. *Adv Mater* 2019; **31**: 1806626.
9. Li M, Dai Y, Ma X et al. Insights into the effect of inner polarization and multiple Ag-O units on high-efficient Ag-based photocatalyst. *Appl Catal B-Environ* 2017; **205**: 211-18.
10. Gelderman K, Lee L, Donne S. Flat-band potential of a semiconductor: using the Mott–Schottky equation. *J Chem Educ* 2007; **84**: 685-88.
11. Hisatomi T, Katayama C, Moriya Y et al. Photocatalytic oxygen evolution using $BaNbO_2N$ modified with cobalt oxide under photoexcitation up to 740 nm. *Energ Environ Sci* 2013; **6**: 3595-99.
12. Maeda K, Domen K. Preparation of $BaZrO_3$-$BaTaO_2N$ solid solutions and the photocatalytic

activities for water reduction and oxidation under visible light. *J Catal* 2014; **310**: 67-74.

13. Hojamberdiev M, Zahedi E, Nurlaela E et al. The cross-substitution effect of tantalum on the visible-light-driven water oxidation activity of $BaNbO_2N$ crystals grown directly by an NH_3-assisted flux method. *J Mater Chem A* 2016; **4**: 12807-17.
14. Maeda K, Lu D, Domen K. Oxidation of Water under Visible-Light Irradiation over Modified $BaTaO_2N$ Photocatalysts Promoted by Tungsten Species. *Angew Chem Int Edit* 2013; **52**: 6488-91.
15. Chen S, Yang J, Ding C et al. Nitrogen-doped layered oxide $Sr_5Ta_4O_{15-x}N_x$ for water reduction and oxidation under visible light irradiation. *J Mater Chem A* 2013; **1**: 5651-59.
16. Ma G, Kuang Y, Murthy DH et al. Plate-like $Sm_2Ti_2S_2O_5$ Particles Prepared by a Flux-Assisted One-Step Synthesis for the Evolution of O_2 from Aqueous Solutions by Both Photocatalytic and Photoelectrochemical Reactions. *J Phys Chem C* 2018; **122**: 13492-99.
17. Townsend TK, Sabio EM, Browning ND et al. Photocatalytic water oxidation with suspended alpha-Fe_2O_3 particles-effects of nanoscaling. *Energ Environ Sci* 2011; **4**: 4270-75.
18. Chen S, Qi Y, Liu G et al. A wide visible-light-responsive tunneled $MgTa_2O_{6-x}N_x$ photocatalyst for water oxidation and reduction. *Chem Commun* 2014; **50**: 14415-17.
19. Fu J, Skrabalak SE. Enhanced Photoactivity from Single-Crystalline $SrTaO_2N$ Nanoplates Synthesized by Topotactic Nitridation. *Angew Chem Int Edit* 2017; **56**: 14169-73.
20. Cui J, Liu T, Qi Y et al. A wide visible light driven complex perovskite $Ba(Mg_{1/3}Ta_{2/3})O_{3-x}N_y$ photocatalyst for water oxidation and reduction. *J Mater Chem A* 2017; **5**: 18870-77.
21. Fujito H, Kunioku H, Kato D et al. Layered perovskite oxychloride Bi_4NbO_8Cl: a stable visible light responsive photocatalyst for water splitting. *J Am Chem Soc* 2016; **138**: 2082-85.
22. Dong B, Cui J, Liu T et al. Development of Novel Perovskite-Like Oxide Photocatalyst $LiCuTa_3O_9$ with Dual Functions of Water Reduction and Oxidation under Visible Light Irradiation. *Adv Energy Mater* 2018; **8**: 1801660.

23. Zhang G, Zang S, Wang X. Layered $Co(OH)_2$ deposited polymeric carbon nitrides for photocatalytic water oxidation. *ACS Catal* 2015; **5**: 941-47.

24. Li J, Cai L, Shang J et al. Giant enhancement of internal electric field boosting bulk charge separation for photocatalysis. *Adv Mater* 2016; **28**: 4059-64.

25. Kanata-Kito T, Matsunaga M, Takakura H et al. Photoreflectance characterization of built-in potential in MBE-produced As-grown GaAs surface. *Proc. SPIE* 1990; **1286**: 56-66.

26. Im JS, Kollmer H, Off J et al. Reduction of oscillator strength due to piezoelectric fields in $GaN/Al_xGa_{1-x}N$ quantum wells. *Phys Rev B* 1998; **57**: R9435.

27. Lefebvre P, Allègre J, Gil B et al. Time-resolved photoluminescence as a probe of internal electric fields in GaN-(GaAl) N quantum wells. *Phys Rev B* 1999; **59**: 15363-67.

28. Morello G, Della Sala F, Carbone L et al. Intrinsic optical nonlinearity in colloidal seeded grown CdSe/CdS nanostructures: Photoinduced screening of the internal electric field. *Phys Rev B* 2008; **78**: 195313.

29. Le Formal F, Sivula K, Grätzel M. The transient photocurrent and photovoltage behavior of a hematite photoanode under working conditions and the influence of surface treatments. *J Phys Chem C* 2012; **116**: 26707-20.
